# Multiobjective Optimization of Chemically Assisted Magnetic Abrasive Finishing (MAF) on Inconel 625 Tubes Using Genetic Algorithm: Modeling and Microstructural Analysis

**DOI:** 10.3390/mi13081168

**Published:** 2022-07-23

**Authors:** Gurpreet Singh, Harish Kumar, Harmesh Kumar Kansal, Kamal Sharma, Raman Kumar, Jasgurpreet Singh Chohan, Sandeep Singh, Shubham Sharma, Changhe Li, Grzegorz Królczyk, Jolanta B. Królczyk

**Affiliations:** 1 Department of Mechanical Engineering, Chandigarh University, Mohali 140413, India; garry.dhiman85@gmail.com (G.S.); harishprashar@gmail.com (H.K.); 2 Department of Mechanical Engineering, University Institute of Engineering and Technology, Chandigarh 160023, India; shaarut@yahoo.com; 3 Institute of Engineering and Technology, GLA University, Mathura 281406, India; kamal.sharma@gla.ac.in; 4 Mechanical Engineering Department, University Center for Research & Development, Chandigarh University, Mohali 140413, India; ramankakkar@gmail.com (R.K.); jaskhera@gmail.com (J.S.C.); 5 Department of Civil Engineering, University Center for Research and Development, Chandigarh University, Mohali 140413, India; drsandeep1786@gmail.com; 6 Department of Mechanical Engineering, Indian Institute of Technology (IIT-Ropar), Rupnagar 140001, India; 7 School of Mechanical and Automotive Engineering, Qingdao University of Technology, Qingdao 266520, China; sy_lichanghe@163.com; 8 Department of Manufacturing and Materials Engineering, Faculty of Mechanical Engineering, Opole University of Technology, Mikolajczyka 5, 45-271 Opole, Poland

**Keywords:** multiresponse optimization, genetic algorithm, chemical-assisted MAF, SEM, Inconel 625, surface finish, material removal (MR)

## Abstract

The demand for the surface integrity of complex structures is drastically increasing in the field of aerospace, marine and automotive industry. Therefore, Inconel alloy, due to its superior attributes, has a wide scope for the improvement in surface integrity. To achieve the precise surface finish and enhance the process performance, process optimization is necessary. In current paper, chemically assisted MAF process parameters were optimized using the genetic algorithm (GA) approach during finishing of Inconel 625 tubes. Regression models were developed for improvement in internal surface finish (PIISF), improvement in external surface finish (PIESF), and material removal (MR) using Design expert software. Then, the surface microstructure of Inconel 625 tubes was analyzed using scanning electron microscopy (SEM). ANOVA analysis predicts that processing time and abrasive size have the highest percentage contribution in improving the surface finish and material removal. Multioptimization results suggested to set the level of processing time (A) at 75 min, surface rotational speed (B) at 60 RPM, weight % of abrasives (C) at 30%, chemical concentration (D) at 500 gm/lt and abrasive size (E) at 40 microns to obtain optimal parameters for PIISF, PIESF and MR responses.

## 1. Introduction

Demand for nickel-based super alloys is drastically increasing in modern industries due to their superior materialistic attributes. They are used in modern applications such as aircraft engines, marine field, gas turbines, petrochemical plants, solar power stations and atomic reactors [1,2]. Moreover, Inconel alloy applications such as spray nozzles used in turbines, cooling channels and hydraulic manifolds are also highlighted by researchers [3]. All the properties are important from a design point of view, but the processing of Inconel 625 super alloy is tough via traditional machining methods due to its high hardness and strength. Ezugwu and Thakur [4] and Gangopadhyay [5] summarized that machinability of Inconel alloy is poor. Various nonconventional machining/finishing methods, namely electric discharge machining (EDM), magnetic abrasive finishing (MAF), abrasive flow machining (AFM) and electrochemical machining (ECM), were recommended by some researchers [6,7,8,9,10].

Among of all, MAF has been extensively used for finishing different inner-geometrical components made of different materials such as aluminum alloy, stainless steel, brass, etc. Shinmura and Yamaguchi [11] tested the viability of MAF for internal finishing of stainless-steel tubes. Yamaguchi and Shinmura [12] improved the internal surface-finish cylindrical-work piece using a pole rotational system. Wang and Hu [13] employed MAF for the finishing of the internal surface of different tubing materials (Aluminum alloy, 316 L SS and brass). Kang and Yamaguchi [14] proposed a multi-pole-tip method to finish the capillary tubes. Nteziyaremyea et al. [15] finished the inner and outer surface simultaneously of stainless-steel needle with MAF. Mulik and Pandey [16] and Misra et al. [17] determined the temperature of the workpiece and brush interface in the MAF process.

**Figure 1:**
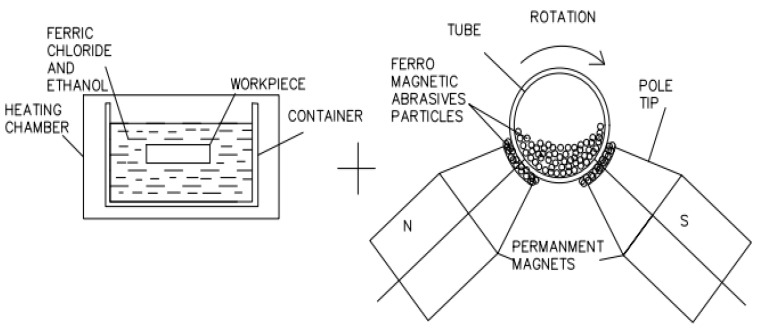
CMAF Principle “Reprinted/adapted with permission from Ref. [15]. Copyright year 2014, copyright owner’s name, Nteziyaremyea et al. [15]”.

However, instead of having many advantages, MAF is less effective at finishing hard materials such as Inconel alloy, tungsten and titanium, due to their superior materialistic characteristics [18,19]. Therefore, chemically assisted magnetic abrasive finishing (CMAF) has recently been developed for machining hard superalloys [20,21]. The CMAF process is the integration of chemical etching and MAF. The processing principle of CMAF is shown in Figure 1. In this process, the tube surface is chemically treated by applying suitable etchants [22,23,24]. Due to a chemical reaction, by-product formation takes place and the workpiece surface becomes diffused [25]. As a result of this surface, intermolecular bonding becomes weak [26]. Further, magnet poles are positioned around the tube, which is chemically treated. Abrasive media is added outside and inside the tube. Abrasive media joins the magnetic force lines when magnetic flux is established.

In the CMAF process, magnetic force attracts the abrasive media towards the internal surface. Outside the tube, abrasive media is impelled against the outer surface of the tube. Whilst the tube is rotated, the inner and outer surface of tube are finished simultaneously.

Du et al. [18] utilized an electrolytic-MAF process on a superalloy (GH4169), and surface roughness descended from 6.37 to 1.77 μm. Judal and Yadava [19] used electrochemical MAF to machine a stainless steel (AISI-304) cylindrical surface and reported that the surface finish was enhanced by increasing the electrolytic current and surface rotational speed. Sihag et al. [20] implemented a chemo-assisted MAF process to finish a tungsten plane surface, and the surface finish was improved by around 79.52% with optimum conditions. Singh et al. [27] used chemical-assisted MAF on Inconel 718 flat surfaces and recommended higher processing time (90 min) and wt% of abrasives (35%) to enhance the MR. Sihag et al. [21] further developed a new hybrid process by integrated ultrasonic vibration, MAF and chemical machining. Experiments were performed on an H2O2 chemical-treated tungsten flat surface and results reported that pulse-on time in ultrasonic vibrations produces extreme impact (21.99%) for altering the surface roughness. At optimum conditions, 86.30% improvement in surface finish was observed. Pandey and Pandey [22] achieved a surface finish of Si-100 wafer of around 18.40 nm with optimum conditions using different chemical oxidizers such as KHSO5, K2S2O8 and H2O2 mixed with alumina slurry (1200 mesh) during polishing using double-disk MAF. Singh et al. improved the internal roundness by around 32% and external roundness by around 26% of Inconel 625 tubes by chemically assisted MAF [23,24]. Singh et al. performed chemically assisted MAF on Inconel 625 tubes and reported that process factors had significant impact on the material removal. The maximum MR 1.02 gms was achieved at a processing time of 75 min, surface rotational speed of 180 RPM, wt% of abrasives 35%, chemical concentration of 600 gm/lt and abrasive size of 60 μm [25].

The researchers utilized various strategies to optimize the MAF process factors to obtain the best results. MAF process parameters were also optimized for surface roughness of stainless steel 202 by using the Taguchi approach [26]. Singh et al. developed regression models for responses such as enhancement in surface finish and MR utilizing ANOVA and response surface methodology [28]. The response surface method was also used to obtain optimal conditions for maximum MR, and the surface finish was also identified via response surface method [29]. Singh et al. performed multiobjective optimization of MAF on EN-31 using the desirability approach with the genetic algorithm [30]. Misra et al. used the genetic algorithm technique for multioptimization of ultrasonic-assisted MAF for surface roughness [31]. Babbar et al. identified optimal MAF variables for surface roughness and MRR by grey relation analysis [32].

In reported work, only the impact of chemically assisted MAF process factors had been analyzed during the finishing of Inconel 625 tubes. To improve process productivity, the optimization of any process is very important. In the present paper, multiobjective optimization of chemically assisted MAF variables for surface finish and material removal was performed using the genetic algorithm approach.

## 2. Experimental Setup

**Figure 2:**
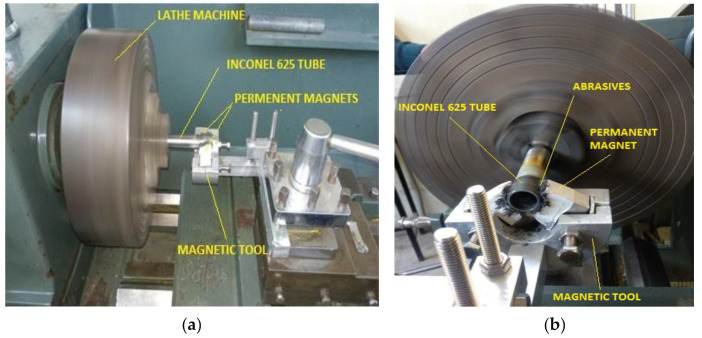
Experimental setup. (**a**) Side view; (**b**) front view.

**Figure 3:**
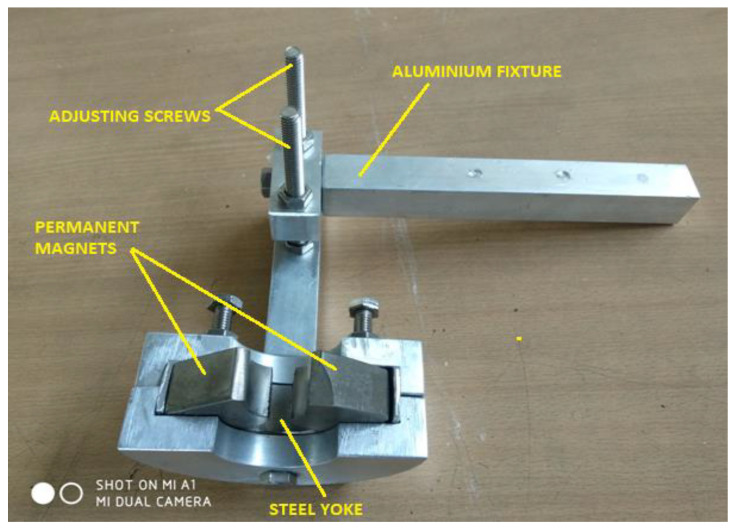
Magnetic tool.

The experimental setup consisted of a magnetic tool that is gripped on the precision lathe machine tool post as represented in Figure 2a,b. Magnetic tool was designed and fabricated as per the requirements of experimental setup. Nd-Fe-B (Neodymium magnet) permanent magnets were mounted on the aluminum fixture. Both magnets were attached with stainless-steel (SS 400) yoke inside the aluminum fixture. Magnetic flux density was found around 0.5 tesla. North (N) and south (S) magnet poles were arranged as per tube diameter toward the tube center. Magnetic poles were insulated with poly-tetra-fluoro-ethylene (PTFE) tape to provide smooth relative motion between N-S poles and rotating tube. Inconel 625 tube was held on the precision lathe machine. Both poles were arranged around the tube with 2 mm gap. Figure 3 shows the magnetic tool used in this experimentation. It consisted of two Nd-Fe-B permanent magnets (Magnet Size-35 mm × 35 mm × 25 mm), stainless-steel (SS-400) yoke, aluminum fixture and stainless-steel adjusting screws. Magnets were mounted on the aluminum fixture and lower poles were attached with stainless-steel (SS-400) yoke. The adjusting screws were used to adjust the height of magnetic tool.

## 3. Methodology

In this investigation, response surface methodology (RSM) is utilized to plan the investigations. RSM is a statistical tool that reduces the number of experiment trails and generates better response for number of parameters. Central composite plan was chosen to plan the experiments. Table 1 represents the CMAF parameters with range.

Before performing the experiments, chemical mixture was prepared with the chosen range of chemical concentration by using combination of ethanol and ferric chloride. In prepared chemical mixture, Inconel 625 tubes were immersed and placed in muffle furnace at 65 °C for 30 min. Further, chemically treated tube was finished using MAF process. After chemical treatment, tube was placed on the precision lathe machine. Then, abrasive media as per selected weight percentage were inserted in the gap between poles and tube in equal amount. Abrasive media were also inserted in the tube. To bind abrasive particles, a barrel-finishing compound was applied on abrasive media. Flexible-magnetic abrasive brush (FMAB) was formed outside and inside the tube by these particles under the magnetic field. This FMAB performed finishing action on the inner and outer surfaces of Inconel 625. After performing the experiment, the surface roughness of internal and external surface was measured by using Telesurf roughness tester. The initial workpiece surface roughness range was 1.01 µm to 4.15 µm for internal surface and 1.23 µm to 4.12 µm for external surface. After the chemical treatment, the surface roughness range was 1.18 µm to 4.28 µm for internal surface and 1.37 µm to 4.31 µm for external surface. After finishing with MAF process, the surface roughness range was 0.43 µm to 2.39 µm for internal surface and 0.84 µm to 2.84 µm for external surface.

Weight of specimen was measured by digital weighing machine to calculate the MR. On the basis of surface roughness readings, the improvement in the internal surface finish (PIISF) and improvement in external surface finish (PIESF) were calculated for each experiment using following relation. Experimental conditions with output characteristics are represented in Table 2.

PIISF/PIESF = (surface roughness of rough surface − surface roughness of finished surface)/surface roughness of rough surface × 100.

## 4. Results and Discussions

The optimization of any process is a very essential criterion to improve the process efficiency. In the present work, multiresponse optimization of chemically assisted MAF process parameters was attempted to obtain the best results in the form of surface finish and material removal by using a genetic algorithm. Analysis of variance (ANOVA) is a promising technique for identifying the significant variables. Table 3 represents the ANOVA results for PIISF. The model *F*-value (5.44) and *p*-value (0.0031) indicate that the model is significant. It has been analyzed that processing time and abrasive size are the major contributing parameters for PIISF. Table 4 represents the ANOVA results for PIESF. The model is significant with the *F*-value (4.64) and *p*-value (0.0061). For PIESF, the processing time and abrasive size are the most significant parameters. Table 5 shows the ANOVA results for MR. The model is significant with *F*-value (10.89) and *p*-value (0.0001). The maximum improvement in MR is achieved by processing time, followed by abrasive size and wt% of abrasives. R-Sq values for PIISF (98%), PIESF (89%) and MR (99%) indicate a strong correlation with experimental results.

**Figure 4:**
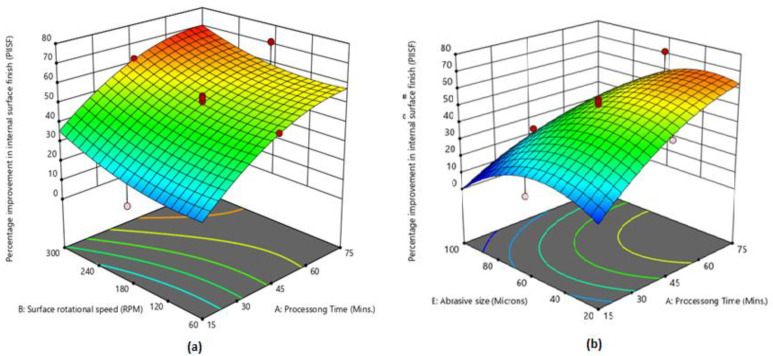
(**a**) Influence of processing time and rotational speed on PIISF; (**b**) influence of abrasive size and chemical concentration on PIISF.

On the basis of regression models, 3D graphs were plotted to analyze the influence of CMAF process factors on the PIISF, PIESF and MR. Figure 4a shows that the maximum PIISF is attained at 75 min processing time and 300 RPM rotational speed. PIISF continuously increases by increasing the processing time. The main reason is that the magnetic tool performs the finishing action for a long duration by increasing the processing time and more peaks are removed from the surface. On other side, PIISF is also increased by enhancing the surface rotational speed. The abrasive particle-striking rate is increased by enhancing the surface rotational speed, which results in more peaks being removed from the workpiece surface [31,32]. Figure 4b shows that PIISF is highest at processing time of 75 min and abrasive size of 40 µm. PIISF is also affected by the use of different abrasive sizes. Initially, PIISF was improved with an increase in abrasive size (up to 40 µm) and afterward started decreasing. It was observed that very fine and very coarse abrasive particles did not produce effective finishing. A very fine abrasive size has small cutting edges that slowly finish the surface, and fewer peaks are removed from the work piece. On the other side, very course particles have large cutting edges that finish rapidly but produce scratches on the surface. Therefore, better improvement in surface finish was achieved using 40 µm and 60 µm.

**Figure 5:**
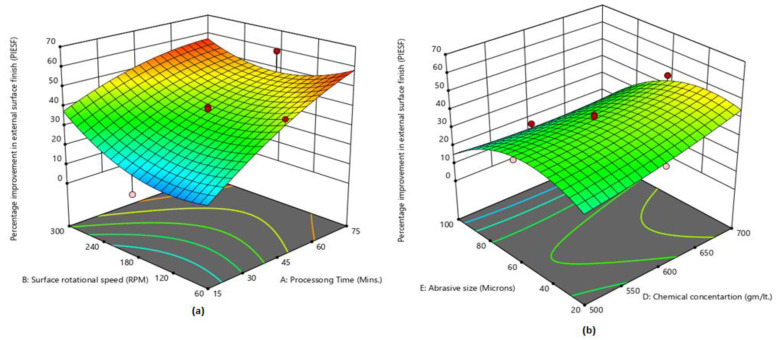
(**a**) Influence of processing time and rotational speed on PIESF; (**b**) influence of abrasive size and chemical concentration on PIESF.

Figure 5a shows that maximum PIESF was attained at a highest processing time of 75 min and 300 RPM rotational speed. PIESF continuously increases with an increase in processing time because the magnetic tool is finishing the surface for a longer duration. On other hand, initially PIESF starts decreasing (up to 120 RPM) and afterward, it starts increasing by enhancing the surface rotational speed. The abrasive particles strike on the workpiece surface very rapidly by enhancing the rotational speed, and the surface-finishing rate is increased, which results in more peaks being reduced from the surface [11,12,13,14,15,16,17,18,19,20,21,22,23,24,27]. Figure 5b shows that PIESF is highest at an abrasive size of 40 µm and a chemical concentration of 700 gm/lt. Initially, PIESF is improved by an increase in abrasive size (till 40 µm) and afterward starts decreasing. In another case, PIESF slowly increases by enhancing the chemical concentration. The chemical reaction becomes faster with the increase in chemical concentration and more uniform etching occurs on the workpiece surface, which results in PIESF being enhanced.

**Figure 6:**
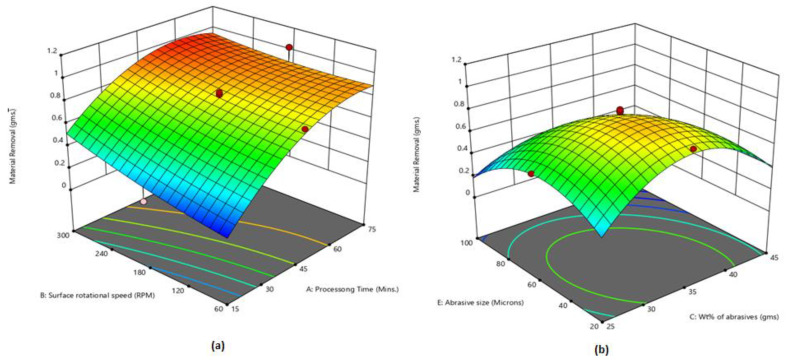
(**a**) Influence of processing time and rotational speed on MR; (**b**) influence of wt% of abrasives and abrasive size on MR.

Figure 6a shows that maximum MR is attained at 60 min processing time and 300 RPM rotational speed. MR is maximized by enhancing the rotational speed and processing time up to a certain limit (60 min). Figure 6b shows that maximum MR is attained using weight % of abrasives at 35% and abrasive size at 40 µm. MR increases by enhancing the weight % up to 35%, then afterwards starts declining. The effective magnetic brush is formed at 35% weight % of abrasives, which is responsible for the improvement in MR. At a lower weight % of abrasives, fewer cutting edges are available in the magnetic brush. At higher weight % of abrasives, the abrasive bonding strength is decreased due to a lower quantity of iron particles, which results in less material being removed from the workpiece surface.

**Figure 7:**
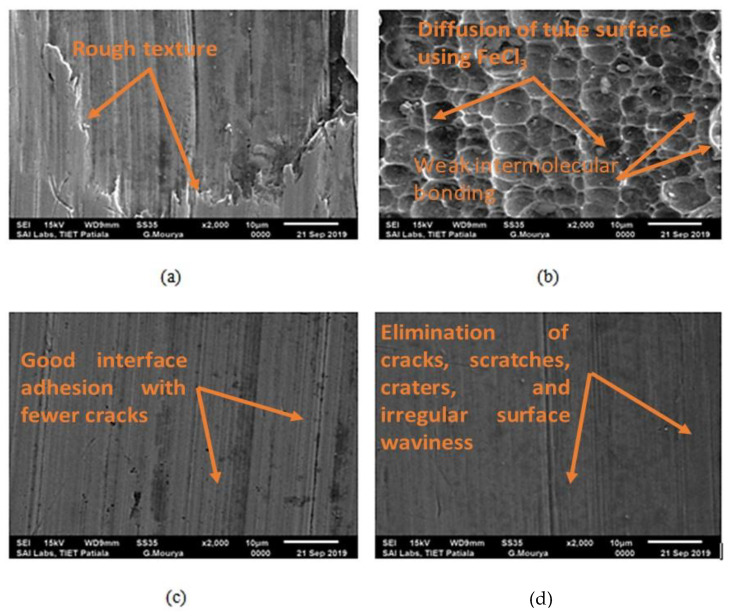
SEM image of Inconel 625 tube. (**a**) Rough surface; (**b**) chemically treated surface; (**c**) finished internal surface (PT-75, AS-60, SRS-180, CC-600, WPS-35%); (**d**) finished external surface (PT-75, AS-60, SRS-180, CC-600, WPS-35%).

The surface microstructure of Inconel 625 tubes was analyzed using scanning electron microscopy (SEM). Figure 7a shows the SEM image of the rough surface of Inconel 625 tubes. The scratches, waviness and craters are clearly visible on the rough surface. Figure 7b shows the SEM image of the chemically treated surface of the Inconel 625 tube. Analysis shows that the tube surface becomes diffused after chemical treatment using FeCl_3_, and intermolecular bonding of the material upper surface becomes weakened. Figure 7c,d show the SEM images of finished internal and external surface samples at a processing time of 75 min. It is clearly visible that the diffused surface, crater and waviness are completely eliminated and scratches are also reduced.

## 5. Multiobjective Optimization

As mentioned in preceding section, the influence of every input variable on output responses is exceptional and nonuniform. Moreover, the variable interaction is significant, and therefore it is tough to pick out best conditions for three output responses. Traditional optimization strategies cannot advise a single set of variables to obtain the best value of PIISF, PIESF and MR for the current work. Thus, there is a requirement to put into impact an advanced optimization method that might also recommend the optimal parametric settings. In present work, regression analysis is implemented, accompanied by the genetic algorithm (GA) and analytical hierarchy methods.

### 5.1. Regression Method

The regression approach is utilized for investigating the influence of input parameters on output responses. Regression analysis is conducted using SPSS software. The current work is targeted on three output responses; consequently, three single-objective equations were created by utilizing the regression approach. Within the production domain, there are various variables that have an impact on the output responses. The relationship between independent and response variables is defined through a mathematical model, which is represented in Equation (1).

(1) $$Y=\beta_{0}+\overset{n\;}{\underset{i=1}{\sum }}\beta_{i\;}\times C_{i\;}+\overset{n\;}{\underset{i=1}{\sum }}\beta_{ii\;}\times C_{i}^{2}+\overset{n\;}{\underset{i=1}{\sum }}\beta_{ij\;}\times C_{i\;}C_{j\;}$$

Equation (1) for five variables can be written as represented in Equation (2), where C1 (processing time), C2 (surface rotational speed), C3 (wt% of abrasives), C4 (chemical concentration) and C5 (abrasive Size) are the process variables and Y indicates the response variables.

(2) $$Y=\beta_{0}+\beta_{1\;}C_{1}+\beta_{2\;}C_{2}+\beta_{3\;}C_{3}+\beta_{4\;}C_{4}+\beta_{5\;}C_{5}+\beta_{6\;}C_{1}\times C_{2}$$

In this work, there are five input CMAF process variables, whereas PIISF, PIESF and MR are the output responses. By using the regression approach, four Equations—(3)–(6)—are generated, whereas maximization of PIISF, PIESF and MR are the main goals, respectively.

3D surface plots show that each CMAF process variable has a nonuniform impact on PIISF, PIESF and MR of finished Inconel 625 tubes. Therefore, there is a requirement to determine a single set of variables that may give the quality outcomes in the form of PIISF, PIESF and MR. This multiobjective issue of CMAF may be resolved by carrying out a genetic algorithm (GA). Expectedly, weighted aggregate methodology, distance function strategy and Taguchi procedures are utilized for multiresponse optimization. However, the precision of these techniques relies on the expertise of a professional who chooses the relative significance and ranking of the objective function. Another obstacle to these methods is the discontinuity of the objective function. Consequently, GA was utilized in the current work for multiobjective problems. GA is a most promising approach that eradicates the necessity of gradient facts and intrinsic parallelism to find the design area. The main objective is to identify a single set of CMAF variables within set limits, which would enhance the PIISF, PIESF and MR. At first, three equations for every objective function (PIISF, PIESF and MR) are changed over into a single equation, as represented in (6).

**PIISF_MAX_** = −233 + 4.54 A − 0.643 B + 9.07 C + 0.024 D + 2.15 E − 0.00960 A *A + 0.000338 B *B − 0.2264 C *C − 0.000114 D *D − 0.01040 E *E − 0.00021 A *B − 0.0158 A *C − 0.00358 A *D − 0.00563 A *E + 0.00854 B *C + 0.000437 B *D + 0.00047 B *E + 0.00925 C *D − 0.0019 C *E − 0.00169 D *E (3)

**PIESF_MAX_** = −81 + 3.70 *A − 0.693 *B + 6.86 *C − 0.268 *D + 1.52 *E − 0.00480 *A *A + 0.000672 *B *B − 0.1532 *C *C + 0.000168 *D *D − 0.00864 *E *E − 0.00153 *A *B − 0.0183 *A *C − 0.00317 *A *D + 0.00083 *A *E + 0.00458 *B *C + 0.000542 *B *D + 0.00125 *B *E + 0.00650 *C *D − 0.0075 *C *E − 0.00125 *D *E (4)

**MR_MAX_** = − 6.07 + 0.0706 A − 0.00816 *B + 0.2217 *C + 0.00372 *D + 0.0291 *E − 0.000230 *A *A + 0.000002 *B *B − 0.004023 *C *C − 0.000002 *D *D − 0.000173* E *E − 0.000019 *A *B + 0.000117 *A *C − 0.000067 *A *D − 0.000017 *A *E + 0.000167 *B *C + 0.000003 *B *D + 0.000024 *B *E + 0.000060 *C *D − 0.000400 *C *E − 0.000006 *D *E (5)

**y** = −0.333 * (−233 + 4.54 *A − 0.643 *B + 9.07 *C + 0.024 *D + 2.15 *E − 0.00960 *A *A + 0.000338 *B *B − 0.2264 *C *C − 0.000114 *D *D − 0.01040 *E *E − 0.00021 *A *B − 0.0158 *A *C − 0.00358 *A *D − 0.00563 *A *E + 0.00854 *B *C + 0.000437 *B *D + 0.00047 *B *E + 0.00925 *C *D − 0.0019 *C *E − 0.00169 *D *E) − 0.333 * (−81 + 3.70 *A − 0.693 *B + 6.86 *C − 0.268* D + 1.52 *E − 0.00480 *A *A + 0.000672 *B *B − 0.1532 *C *C + 0.000168 *D *D − 0.00864 *E *E − 0.00153 *A *B − 0.0183 *A *C − 0.00317 *A *D + 0.00083 *A *E + 0.00458 *B *C + 0.000542 *B *D + 0.00125 *B *E + 0.00650 *C *D − 0.0075 *C *E − 0.00125 *D *E) − 0.333* (−6.07 + 0.0706* A − 0.00816 *B + 0.2217 *C + 0.00372 *D + 0.0291 *E − 0.000230 *A *A + 0.000002 *B *B − 0.004023 *C *C − 0.000002 *D *D − 0.000173* E *E − 0.000019 *A *B + 0.000117 *A *C − 0.000067 *A *D − 0.000017 *A *E + 0.000167 *B *C + 0.000003 *B *D + 0.000024 *B *E + 0.000060 *C *D − 0.000400 *C *E 0.000006*D *E (6)

### 5.2. Genetic Algorithm (GA)

**Figure 8:**
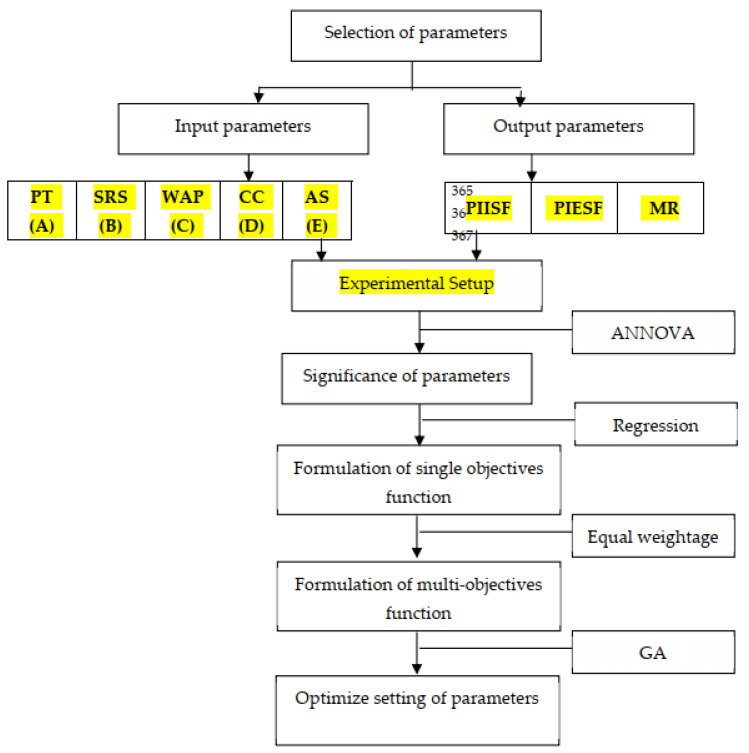
Flow chart used for multiobjective optimization.

GA is utilized to resolve nonlinear objective functions. GA was developed by Holland [33], and in 1989, Goldberg [34] reformed it. GA is utilized to accomplish the best fitness values, and its outcomes are very authentic, particularly in the problem of constrained optimization. In present study, a total of five input parameters and three output parameters were considered for the design of the experiment. Previous studies have proved that soft computing techniques provide more reliable optimum results in the case of multiobjective optimization [33,34,35,36,37,38,39,40,41,42,43]. GA is an inbuilt tool in MATLAB, which make it friendlier for users. Moreover, GA is the most commonly used technique for optimization problems. As per the SCOPUS database, more than sixty-four thousand articles have been published to date. Researchers have successfully implemented GA in various disciplines, such as machining [35], drug repurposing [36], automobiles [37], earth work activities [38], transportation [39], antenna [40], energy planning [41], electric vehicle [42], structural design [43], etc. Figure 8 represents the genetic algorithm flow chart.

The genetic algorithm was performed using MATLAB, and optimization results are represented in Table 6. The multiresponse optimization solution indicated that the values of PT, SRS and CC are different for PIISF but remain constant for PIESF and MR in optimization solutions. Moreover, the values of WAP and AS are different for all optimization conditions.

**Figure 9:**
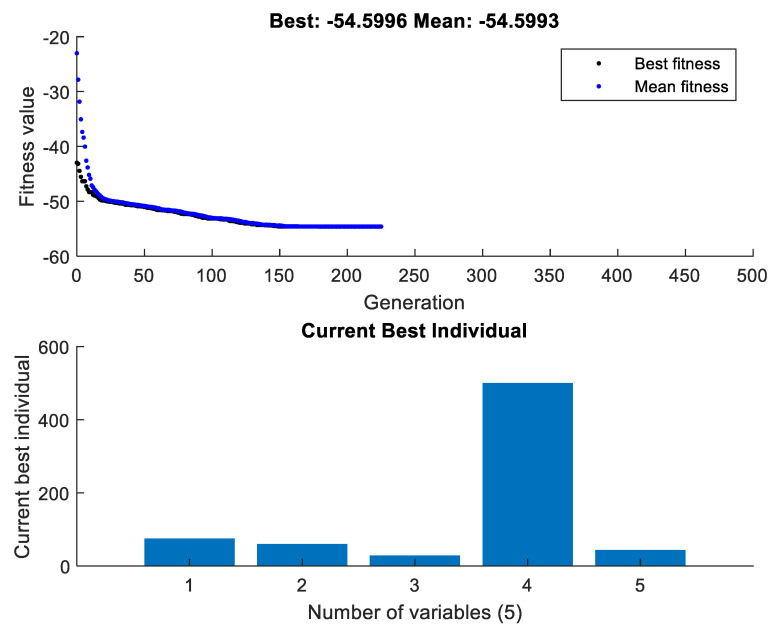
Current best individual using GA.

For multiobjective optimization, the best value of each variable is represented in Figure 9. It tended to be found that approximately 230 generations were involved to find the best optimization results when GA codes were performed utilizing MATLAB. Moreover, the best fitness and mean values are extremely near one another, which confirms the precision of the results. It is suggested that processing time must be set at 75 min, surface rotational speed at 60 RPM, wt% of abrasives at 28.51% (approx. 30%), the chemical concentration at 500 gm/lt and abrasive size at 43.98 (approx. 40 microns).

## 6. Conclusions

In this work, multiresponse optimization of chemically assisted MAF variables for PIISF, PIESF and MR was performed using a genetic algorithm approach. The following points are summarized from this optimization work.

- The developed regression model shows a strong correlation with the experimental results. The processing time and abrasive size are the major significant parameters for PIISF, PIESF and MR.
- The SEM images of the rough surface of Inconel 625 tubes exhibiting scratches, waviness and craters are clearly visible on the rough surface. Moreover, the SEM image of the chemically treated surface of the Inconel 625 tube reported that the tube surface became diffused after chemical treatment using FeCl_3_ and that the intermolecular bonding of the material’s upper surface became weakened.
- Multioptimization results using a genetic algorithm suggested that PIISF, PIESF and MR are best optimized by setting the value of processing time (A) at 75 min, surface rotational speed (B) at 60 RPM, weight % of abrasives (C) at 30%, chemical concentration (D) at 500 gm/lt and abrasive size (E) at 40 microns.

## Figures and Tables

**Table 1:** CMAF parameters.

CMAF Parameters	Units	Symbol	Levels				
			−2	−1	0	1	2
1. Processing Time (PT)	Mins.	A	15	30	45	60	75
2. Surface Rotational Speed (SRS)	RPM	B	60	120	180	240	300
3. Weight % of Abrasive Particles (WAP)	gms.	C	25	30	35	40	45
4. Chemical Concentration (CC)	gm./Lt.	D	500	550	600	650	700
5. Abrasive size (AS)	microns	E	20	40	60	80	100
**Other Parameters**							
Workpiece	Material—Inconel 625 Dimensions—(Ø25 × 150 × 2 mm)						
Permanent Magnet	Material—Nd-Fe-B size—(35 mm × 35 mm × 25 mm) SS 400 Steel yoke						
Amount of SiC (Abrasive) and Iron Particles	3 gms for each pole						
Size of Iron Particle	300 µm						
Lubricant	Barrel-finishing compound (Ashfa Coorporation, Mumbai, Maharashtra)						
Pole work gap	Gap = 2 mm						
Etchant	FeCl_3_ diluted with Ethanol						
Etching Time	Time = 30 min						
Etching Temperature	Temperature = 65 °C						
**Response Factors**							
1. Improvement in internal surface finish (PIISF)							
2. Improvement in external surface finish (PIESF)							
3. Material removal (MR)							

**Table 2:** Experimental results with responses.

S. No.	Input Process Parameters					Output Responses		
	PT (A)	SRS (B)	WAP (C)	CC (D)	AS (E)	PIISF	PIESF	MR
1	30	120	40	550	40	24	23	0.34
2	60	120	40	550	80	26	24	0.37
3	30	240	40	550	80	25	20	0.3
4	45	180	35	600	100	23	16	0.26
5	45	180	35	600	60	52	39	0.74
6	60	120	30	650	80	32	28	0.64
7	30	120	30	650	40	42	33	0.71
8	30	240	40	650	40	52	45	0.79
9	60	120	30	550	40	61	49	0.88
10	15	180	35	600	60	12	8	0.19
11	30	240	30	650	80	32	29	0.64
12	60	240	30	650	40	58	44	0.8
13	45	180	45	600	60	25	19	0.28
14	45	180	25	600	60	30	26	0.54
15	45	180	35	600	60	45	31	0.74
16	75	180	35	600	60	71	59	1.02
17	45	300	35	600	60	62	50	0.9
18	45	180	35	600	60	43	39	0.86
19	45	180	35	600	20	44	32	0.81
20	30	120	40	650	80	21	14	0.22
21	60	240	30	550	80	45	39	0.71
22	45	180	35	600	60	53	34	0.84
23	60	240	40	650	80	38	32	0.64
24	45	60	35	600	60	48	45	0.79
25	45	180	35	700	60	51	46	0.84
26	45	180	35	500	60	47	33	0.75
27	45	180	35	600	60	54	39	0.85
28	45	180	35	600	60	51	40	0.84
29	30	240	30	550	40	36	29	0.51
30	30	120	30	550	80	28	18	0.36
31	60	240	40	550	40	51	37	0.83
32	60	120	40	650	40	46	38	0.76

**Table 3:** ANOVA for PIISH.

Source	Sum of Squares	Degree of Freedom	Mean Square	*F*-Value	*p*-Value	Remarks
**Model**	5548.91	20	277.45	5.44	0.0031	Significant
**A—Processing time**	1926.04	1	1926.04	37.76	<0.0001	
**B—Surface rotational Speed**	301.04	1	301.04	5.9	0.0334	
**C—Wt% of abrasives**	155.04	1	155.04	3.04	0.1091	
**D—Chemical concentration**	45.38	1	45.38	0.8896	0.3659	
**E—Abrasive size**	1134.38	1	1134.38	22.24	0.0006	
**AB**	0.5625	1	0.5625	0.011	0.9183	
**AC**	22.56	1	22.56	0.4423	0.5197	
**AD**	115.56	1	115.56	2.27	0.1604	
**AE**	45.56	1	45.56	0.8932	0.3649	
**BC**	105.06	1	105.06	2.06	0.1791	
**BD**	27.56	1	27.56	0.5404	0.4777	
**BE**	5.06	1	5.06	0.0992	0.7586	
**CD**	85.56	1	85.56	1.68	0.2218	
**CE**	0.5625	1	0.5625	0.011	0.9183	
**DE**	45.56	1	45.56	0.8932	0.3649	
**A²**	136.74	1	136.74	2.68	0.1298	
**B²**	43.37	1	43.37	0.8502	0.3763	
**C²**	939.41	1	939.41	18.42	0.0013	
**D²**	2.37	1	2.37	0.0464	0.8334	
**E²**	507.41	1	507.41	9.95	0.0092	
**Residual**	561.09	11	51.01			
**Lack of fit**	457.76	6	76.29	3.69	0.0866	not significant
**Pure error**	103.33	5	20.67			
**Cor total**	6110	31				

R^2^ = 0.98; std. dev. = 7.14; mean = 41.50; C.V.% = 17.21; adeq. precision = 7.50.

**Table 4:** ANOVA for PIESH.

Source	Sum of Squares	Degree of Freedom	Mean Square	*F*-Value	*p*-Value	Remarks
**Model**	3747.27	20	187.36	4.64	0.0061	significant
**A—Processing time**	1380.17	1	1380.17	34.15	0.0001	
**B—Surface rotational Speed**	140.17	1	140.17	3.47	0.0895	
**C—Wt% of abrasives**	104.17	1	104.17	2.58	0.1367	
**D—Chemical concentration**	104.17	1	104.17	2.58	0.1367	
**E—Abrasive size**	661.5	1	661.5	16.37	0.0019	
**AB**	30.25	1	30.25	0.7484	0.4055	
**AC**	30.25	1	30.25	0.7484	0.4055	
**AD**	90.25	1	90.25	2.23	0.1632	
**AE**	45.56	1	45.56	0.0247	0.8779	
**BC**	30.25	1	30.25	0.7484	0.4055	
**BD**	42.25	1	42.25	1.05	0.3285	
**BE**	36	1	36	0.8907	0.3656	
**CD**	42.25	1	42.25	1.05	0.3285	
**CE**	9	1	9	0.2227	0.6462	
**DE**	25	1	25	0.6185	0.4482	
**A²**	34.19	1	34.19	0.8458	0.3775	
**B²**	171.85	1	171.85	4.25	0.0636	
**C²**	430.19	1	430.19	10.64	0.0076	
**D²**	5.19	1	5.19	0.1283	0.727	
**E²**	350.06	1	350.06	8.66	0.0134	
**Residual**	444.61	11	40.42			
**Lack of fit**	378.61	6	63.1	4.78	0.0535	not significant
**Pure error**	66	5	13.2			
**Cor total**	4191.88	31				

R^2^ = 0.89; std. dev. = 6.36; mean = 33.06l C.V.% = 19.23l; adeq. precision = 7.54.

**Table 5:** ANOVA for MR.

Source	Sum of Squares	Degree of Freedom	Mean Square	*F*-Value	*p*-Value	Remarks
**Model**	1.63	20	0.0813	10.89	0.0001	significant
**A—Processing time**	0.4874	1	0.4874	65.29	<0.0001	
**B—Surface rotational speed**	0.0561	1	0.0561	7.51	0.0192	
**C—Wt% of abrasives**	0.0963	1	0.0963	12.9	0.0042	
**D—Chemical concentration**	0.0486	1	0.0486	6.51	0.0269	
**E—Abrasive size**	0.3361	1	0.3361	45.02	<0.0001	
**AB**	0.0049	1	0.0049	0.6564	0.435	
**AC**	0.0012	1	0.0012	0.1641	0.6932	
**AD**	0.04	1	0.04	5.36	0.0409	
**AE**	0.0004	1	0.0004	0.0536	0.8212	
**BC**	0.04	1	0.04	5.36	0.0409	
**BD**	0.0012	1	0.0012	0.1641	0.6932	
**BE**	0.0132	1	0.0132	1.77	0.2101	
**CD**	0.0036	1	0.0036	0.4823	0.5018	
**CE**	0.0256	1	0.0256	3.43	0.091	
**DE**	0.0006	1	0.0006	0.0837	0.7777	
**A²**	0.0788	1	0.0788	10.55	0.0078	
**B²**	0.002	1	0.002	0.2631	0.6182	
**C²**	0.2967	1	0.2967	39.74	<0.0001	
**D²**	0.0005	1	0.0005	0.0733	0.7916	
**E²**	0.1409	1	0.1409	18.88	0.0012	
**Residual**	0.0821	11	0.0075			
**Lack of fit**	0.0664	6	0.0111	3.53	0.0938	not significant
**Pure error**	0.0157	5	0.0031			
**Cor total**	1.71	31				

R2 = 0.99; std. dev. = 0.086; mean = 0.65; C.V.% = 13.32; adeq. precision = 10.98.

**Table 6:** Multiresponse optimization outcomes using genetic algorithm.

Response Parameters	Optimization	PT (A)	SRS (B)	WAP (C)	CC (D)	AS (E)	Objective Function
**PIISF_MAX_**	**Single-Objective**	61.95	300.00	37.69	700.00	33.05	77.81
**PIESF_MAX_**	**Single-Objective**	75.00	60.00	28.25	500.00	47.47	80.65
**MR_MAX_**	**Single-Objective**	75.00	60.00	31.66	500.00	39.31	1.10
	**Multiobjective**	75.00	60.00	28.51	500.00	43.98	54.60

## Data Availability

No data were used to support this study.
